# Are health care workers (HCWs) of long term care facilities (LTC) ready to take care of HIV older patients?

**DOI:** 10.1186/1471-2334-14-S2-P44

**Published:** 2014-05-23

**Authors:** Philippe AL Henrivaux, Yvette RMG Fairon

**Affiliations:** 1 St Joseph Hospital, Liège, Belgium; 2 St Luc University Hospital, Brussels, Belgium

## Objectives

We have the feeling that the problem to find a place for elderly patients in long term facilities is even worse for those who are HIV+. This is probably due to the fact that HCWs in LTC are not sufficiently aware of the potential presence of HIV in their professional life and that they do not have enough experience of this type of care.

## Methods

We contacted 32 LTC with which we had never got in touch before. Initially, we phoned the nurses' responsibles and asked to spontaneously comment the demand of a place for a HIV+ old patient. Thereafter, we sent a written questionnaire about their feelings of having such a patient in their establishment.

## Results

All of them (32/32) said that they have no experience with HIV patients and they want preliminary information. A quarter of them (8/32) never thought about HIV in their professional life. 3/32 evoked the problem as they organized the care for a HCV patient. Concerning the written questionnaire, we received answers from 15/32 institutions. Based on their responses, we tried to assign a note (mean+/-SD: 8.2+/-2.1) about their feelings concerning the care of HIV patients. Two institutions got a note above the mean+1SD, suggesting that they are more ready to take care of a HIV patient.

## Conclusions

HCWs from rest homes in our region are not sufficiently informed and trained to welcome HIV older patients. They want preliminary information. We think also that a practical experience is the best way in order to tackle this health problem. Therefore, we invite them to participate to the care of HIV patients in our service.

